# Quercetin Attenuates Metabolic Dysfunction and Reshapes Hypothalamic m6A-Related, Leptin–Insulin, and Melanocortin-Associated Transcription in High-Fat-Diet-Fed Rats

**DOI:** 10.3390/metabo16100773

**Published:** 2026-10-13

**Authors:** Alexis Alejandro García-Rivero, Ángel Miliar-García, Antonio Ávila-Guerrero, María de los Ángeles Martínez-Godínez, María del Carmen Castillo-Hernández, Gustavo Guevara-Balcázar, Aarón Domínguez-López

**Affiliations:** 1 Laboratorio de Biología Molecular, Sección de Estudios de Posgrado e Investigación, Escuela Superior de Medicina, Instituto Politécnico Nacional, Mexico City 11340, Mexico; agarciar2231@alumno.ipn.mx (A.A.G.-R.); amillarg@ipn.mx (Á.M.-G.); aavilag2300@alumno.ipn.mx (A.Á.-G.); mmartinezgo@ipn.mx (M.d.l.Á.M.-G.); 2 Laboratorio de Farmacología y Medicina Hiperbárica Experimental, Sección de Estudios de Posgrado e Investigación, Escuela Superior de Medicina, Instituto Politécnico Nacional, Mexico City 11340, Mexico; ccastillohe@ipn.mx (M.d.C.C.-H.); gguevarab@ipn.mx (G.G.-B.)

**Keywords:** quercetin, high-fat diet, hypothalamus, m6A, FTO, leptin–insulin-associated transcription, melanocortin-associated transcription, RT-qPCR, metabolic dysfunction, transcriptional remodeling

## Abstract

**Background/Objectives:** High-fat diet (HFD) feeding disrupts systemic metabolism and hypothalamic mechanisms that coordinate energy balance. Whether quercetin modifies coordinated hypothalamic transcription linking m6A-related regulation with leptin–insulin- and melanocortin-associated biology remains unclear. We examined the metabolic and hypothalamic transcriptional response to quercetin during chronic HFD exposure. **Methods:** Male Wistar rats were studied for 16 weeks in a 2 × 2 factorial design: Control (*n* = 8), Control + quercetin (*n* = 5), HFD (*n* = 5), and HFD + quercetin (*n* = 6). Quercetin was administered orally at 100 mg/kg/day. Body weight, fasting glucose, food/fluid intake, calculated energy intake, oral glucose tolerance, terminal adiposity, lipids, and a 21-transcript hypothalamic RT-qPCR panel were evaluated. Analyses included factorial models with HC3 inference and FDR control, PCA/PERMANOVA, predefined transcriptional modules, bootstrap and leave-one-sample-out sensitivity analyses, treatment-adjusted covariance, and shrinkage regularization. **Results:** At week 16, HFD + quercetin reduced glucose AUC relative to HFD by 2415 mg·min/dL (q = 0.0029), visceral fat by 5.16 g (q = 0.004), and triglycerides by 47.03 mg/dL (q < 0.001), without a corresponding difference in final body weight. Diet × quercetin interactions were supported for week-16 AUC, visceral adiposity, triglycerides, and 15 of 21 hypothalamic transcripts. Sixteen of 21 transcripts met robustness criteria for the HFD + quercetin versus HFD contrast. PC1 and PC2 captured 73.5% of transcriptional variance, and all three predefined transcriptional modules showed robust diet-dependent responses. Regularized covariance sensitivity preserved the direction of all 210 treatment-adjusted transcript associations. **Conclusions:** The principal metabolic effects of quercetin were predominantly evident under HFD conditions and were accompanied by coordinated hypothalamic transcriptional remodeling across m6A-related, leptin–insulin-associated, and melanocortin-associated domains. These data define a transcriptional architecture for mechanistic follow-up without implying direct measurement of circulating leptin or insulin, m6A activity, protein abundance, or pathway activation.

## 1. Introduction

High-fat feeding alters metabolic regulation through peripheral and central mechanisms. The hypothalamus is particularly relevant because it integrates hormonal and nutrient-related signals that shape energy balance, glucose regulation, and feeding behavior [1,2]. Experimental exposure to high-fat diets promotes inflammatory responses within the hypothalamus and can impair central leptin and insulin responsiveness, particularly within the mediobasal hypothalamus and arcuate nucleus [2,3].

Leptin and insulin converge on intracellular systems that include IRS-PIK3R1-AKT-FOXO1 signaling, while SOCS3 participates in negative regulation of leptin action [4,5,6]. POMC and AGRP/NPY neurons respond to these metabolic cues and form a central component of the melanocortin system, in which MC4R contributes to downstream control of food intake and energy expenditure [7,8]. High-fat feeding can alter the transcription of appetite-related genes in the hypothalamus, including Lepr and neuropeptide-associated targets [9].

RNA N6-methyladenosine (m6A) adds another regulatory layer to metabolic gene control. The METTL3-METTL14 methyltransferase complex and associated proteins such as WTAP, RBM15/RBM15B, VIRMA, and ZC3H13 participate in m6A deposition; FTO and ALKBH5 remove the modification, whereas YTH-domain proteins recognize m6A-marked RNA and influence RNA fate [10]. Altered expression or activity of these regulators has been linked to glucose and lipid metabolism and to metabolic disease [10,11].

The relationship between m6A-associated regulation and hypothalamic metabolic control is becoming increasingly specific. In mice exposed to prolonged HFD feeding, hypothalamic FTO contributed to leptin resistance through an m6A-dependent CX3CL1 mechanism, and FTO deficiency in leptin-receptor-expressing neurons partially attenuated the phenotype [12]. This work provides experimental support for direct molecular coupling between an m6A regulator and hypothalamic leptin responsiveness under obesogenic conditions.

Quercetin has been investigated in experimental models of diet-induced metabolic dysfunction. In HFD-fed rats, quercetin reduced adipocyte hypertrophy and attenuated insulin resistance and inflammatory changes [13]. Other rodent studies have reported improved glucose tolerance and lower circulating triglyceride and cholesterol concentrations [14]. A central response has also been described: quercetin reduced hypothalamic inflammatory cytokines and microglial activation in HFD-fed obese mice [15].

From a human nutrition perspective, the metabolic relevance of quercetin remains of interest, although clinical evidence has been heterogeneous. Randomized controlled trials and meta-analyses in humans have generally not demonstrated consistent effects on body weight, fasting glucose, or insulin resistance, although modest improvements in selected cardiometabolic outcomes have been reported under specific doses, durations, or clinical conditions [16,17,18]. Importantly, a randomized controlled trial in overweight-to-obese individuals found no significant effects of quercetin supplementation on circulating leptin or several systemic metabolic markers [19]. This heterogeneity suggests that the metabolic effects of quercetin may not be adequately captured by anthropometric outcomes alone and raises the possibility that tissue-specific regulatory mechanisms contribute to inter-individual differences in response. In this context, experimental models can provide mechanistic information that cannot be readily obtained from nutritional intervention studies in humans, particularly regarding hypothalamic transcriptional regulation and its potential integration with m6A, leptin–insulin, and melanocortin signaling.

These observations raise the possibility that quercetin-associated metabolic improvement is accompanied by broader changes in hypothalamic transcription. The present study therefore examined male Wistar rats exposed to HFD with or without quercetin and evaluated both metabolic phenotype and a 21-gene hypothalamic panel spanning m6A-related regulation, leptin–insulin-associated transcription, melanocortin-associated transcription, and oxidative response. The analysis was designed to test individual transcripts and coordinated transcriptional structure through multivariate, module-level, and treatment-adjusted covariance approaches.

## 2. Materials and Methods

### 2.1. Animals and Ethical Approval

Adult male Wistar rats were group-housed in standard laboratory cages with bedding under controlled environmental conditions (12 h light/12 h dark cycle, 22 ± 2 °C, and 40–60% relative humidity) with free access to food and water. Experimental procedures followed institutional guidelines for the care and use of laboratory animals and were approved by the Bioethics Committee of the Escuela Nacional de Medicina y Homeopatía, Instituto Politécnico Nacional (approval no. CBE/005/2021). Animals entered the protocol at 8 weeks of age and were followed for 16 weeks. Animal-welfare considerations were incorporated according to the principles of Replacement, Reduction, and Refinement. Replacement was considered in relation to the study objective, which required an intact in vivo model to evaluate integrated metabolic and hypothalamic responses to chronic dietary exposure and quercetin treatment that could not be adequately reproduced in an isolated in vitro system. Reduction was addressed through the 2 × 2 factorial design and longitudinal phenotyping, allowing multiple metabolic and molecular outcomes to be obtained from the same animals rather than from separate experimental cohorts. Refinement included group housing under controlled conditions, free access to food and water, standardized handling, and deep anesthesia before terminal blood and tissue collection.

### 2.2. Experimental Design and Quercetin Administration

The experiment used a 2 × 2 factorial design defined by diet (Control or HFD) and quercetin treatment (No or Yes). Before the intervention, animals were randomly assigned to the four experimental groups using a computer-generated randomization sequence. The allocation sequence was concealed until each animal had been assigned to an experimental group. Investigators responsible for metabolic phenotyping, tissue collection, RT-qPCR, and statistical analysis were blinded to group allocation. Animals and biological samples were identified using coded identifiers throughout data acquisition, laboratory processing, and analysis. Group identities were revealed only after completion of the prespecified analyses.

A total of 26 animals entered the experimental protocol and were initially allocated to Control (n = 8), Control + quercetin (*n* = 6), HFD (*n* = 6), and HFD + quercetin (n = 6). A slightly larger Control group was predefined to provide a broader reference group for comparisons across the three experimental conditions. Two animals died during follow-up: one in the Control + quercetin group at week 8 and one in the HFD group at week 11. Consequently, 24 animals completed the experimental protocol, resulting in final analytical group sizes of Control (*n* = 8), Control + quercetin (*n* = 5), HFD (n = 5), and HFD + quercetin (*n* = 6). These losses occurred during the experimental period and were not exclusions based on metabolic, molecular, or statistical outcomes.

Control animals received Laboratory Rodent Diet 5001 (LabDiet, St. Louis, MO, USA), with a physiological fuel value of approximately 3.35 kcal/g and a manufacturer-reported energy distribution of approximately 28.9% from protein, 13.6% from fat, and 57.5% from carbohydrate. The HFD condition consisted of a hypercaloric high-fat/high-carbohydrate formulation prepared from approximately 50% (*w*/*w*) powdered standard rodent chow, 20% vegetable shortening, 15% vegetable oil, and 15% corn syrup. The energy density used for the experimental HFD was approximately 5.05 kcal/g; based on the formulation and study energy factors, the estimated energy distribution was approximately 9.6% from protein, 66.9% from fat, and 23.5% from carbohydrate. During experimental weeks 0–1, animals assigned to the HFD groups received 10% (*w*/*v*) fructose in the drinking fluid as part of the induction protocol; thereafter, plain drinking water was provided. Quercetin (≥95% purity by HPLC; Sigma-Aldrich, Q4951, St. Louis, MO, USA) was freshly suspended in 0.5% sodium carboxymethylcellulose (Na-CMC) before administration and protected from direct light until use. It was administered by oral gavage at 100 mg/kg/day throughout the intervention, at a standardized time during the light phase; non-quercetin groups received an equivalent volume of the same vehicle. No separate analytical stability study was performed; preparation immediately before dosing was used to minimize storage-related degradation. The dose represents an experimental efficacy exposure within the range used in rodent metabolic studies [13,14,15,20,21,22,23] and was not intended to model usual human dietary quercetin intake. The experimental timeline and molecular panel are summarized in Figure 1.

### 2.3. Longitudinal Metabolic Phenotyping and Oral Glucose Tolerance Testing

Body weight and fasting blood glucose were recorded longitudinally from baseline to week 16. Because animals were group-housed, food and fluid intake were recorded at the cage/group level and are presented descriptively rather than treated as independent animal-level observations. Calculated energy intake incorporated the energy contribution of the solid diet; during weeks 0–1, the caloric contribution of the 10% fructose drinking solution was additionally included. Weekly group-level food, fluid, and energy-intake values are provided in Supplementary Table S5. OGTTs were performed at baseline and at weeks 8 and 16. Animals underwent a standardized 12 h overnight fast spanning the dark phase and ending in the early light phase, after which glucose was administered orally at 2 g/kg body weight. Tail-blood glucose was measured immediately before the glucose load (0 min) and at 30, 60, 90, and 120 min. Total area under the curve (AUC) and incremental AUC (iAUC) were calculated from the serial glucose measurements.

### 2.4. Terminal Tissue Collection and Lipid Measurements

At week 16, animals were deeply anesthetized with sodium pentobarbital (Pentobarbital Inyectable, 65 mg/mL; Laboratorios Aranda, S.A. de C.V., Querétaro, Querétaro, Mexico; 150 mg/kg, intraperitoneally) and euthanized by exsanguination after loss of consciousness. Terminal procedures were conducted using a standardized, interleaved collection order across experimental groups to minimize confounding by time of day. Retroperitoneal adipose tissue, used as the visceral adiposity measure, whole brain, and hypothalamic tissue were collected and weighed. Blood was obtained for terminal biochemical assessment. Total cholesterol, triglycerides, and high-density lipoprotein cholesterol (HDL-C) were measured using the Mission® Cholesterol Meter with Mission^®^ lipid-panel test devices (ACON Laboratories, Inc., San Diego, CA, USA), a portable reflectance-photometry-based lipid analyzer. Low-density lipoprotein cholesterol (LDL-C) was the device-calculated value generated from the measured lipid profile rather than a direct measurement. Concentrations are reported in mg/dL. Circulating leptin and insulin concentrations were not measured in this experiment.

### 2.5. Hypothalamic RNA Isolation and cDNA Synthesis

Hypothalamic tissue was dissected immediately after brain removal, rapidly frozen, and stored at −80 °C until molecular analysis. Total RNA was isolated from individual samples using TRIzol™ Reagent (Invitrogen, Thermo Fisher Scientific, Carlsbad, CA, USA) by phenol-chloroform extraction. Tissue was homogenized in TRIzol, followed by chloroform-induced phase separation, recovery of the aqueous phase, isopropanol precipitation, washing with 75% ethanol, and resuspension in RNase-free water. RNA concentration and purity, including A260/A280 and A260/A230 ratios, were assessed using a NanoDrop™ 2000 spectrophotometer (Thermo Fisher Scientific, Wilmington, DE, USA). Ratios close to 2.0 were considered consistent with acceptable spectrophotometric purity; operationally, values of approximately 1.8–2.2 for both A260/A280 and A260/A230 were accepted for downstream cDNA synthesis. Complementary DNA was synthesized from total RNA using M-MLV Reverse Transcriptase (Invitrogen, Thermo Fisher Scientific, Carlsbad, CA, USA)according to the manufacturer’s instructions.

### 2.6. Quantitative Real-Time PCR and Transcriptional Panel

Quantitative real-time PCR was performed using Rotor-Gene Q system (QIAGEN GmbH, Hilden, Germany) with SYBR Green qPCR Master Mix (No ROX; MedChemExpress, HY-K0523, Monmouth Junction, NJ, USA). Reactions were prepared in a final volume of 10 µL using gene-specific forward and reverse primers and cDNA as template. No-template controls were included for each primer pair. Amplification used assay-specific conditions; primer sequences, NCBI RefSeq accessions, and assay-specific annealing temperatures are provided in Appendix A, Table A1. Relative transcript abundance was normalized to β-actin (ACTB) and calculated by the comparative 2^−ΔΔCt^ method using the Control group as the calibrator. Statistical modeling used log_2_(2^−ΔΔCt^), which provides a symmetric scale for increases and decreases in relative transcript abundance. The PIK3R1 assay targeted Rattus norvegicus Pik3r1 (RefSeq NM_013005.3) using the primer pair listed in Table A1 and is therefore referred to as PIK3R1 throughout. Retained quality-control records for FTO and ACTB included technical triplicates, dilution-series standard curves, amplification and melt curves, and no-template controls. FTO and ACTB amplification efficiencies were 96.4% and 98.6%, respectively (R^2^ = 0.9983 and 0.9987), and median within-sample triplicate SDs were 0.062 and 0.061 cycles, respectively. Melt profiles showed tightly clustered mean melting temperatures of approximately 81.65 °C for FTO and 84.21 °C for ACTB, and no Cq was recorded for the corresponding no-template controls. Detailed FTO/ACTB assay-quality data are provided in Supplementary Table S6 and Figure S4.

All animals completing the experimental protocol contributed one independent biological replicate to the RT-qPCR analyses (Control, n = 8; Control + quercetin, n = 5; HFD, n = 5; HFD + quercetin, n = 6). The same set of biological samples was analyzed across all 21 target transcripts and ACTB.

The 21-target panel comprised FTO, ALKBH5, METTL3, METTL14, WTAP, RBM15, RBM15B, VIRMA, ZC3H13, and YTHDC1 for m6A-related regulation; LEPR, IRS1, PIK3R1, AKT2, SOCS3, and FOXO1 for leptin–insulin-associated transcription; POMC, NPY, AGRP, and MC4R for melanocortin-associated transcription; and SOD2 as an oxidative-response transcript. Molecular results are interpreted as differences in mRNA abundance and not as direct measurements of protein abundance, enzymatic activity, pathway activation, circulating hormone concentrations, or global m6A levels.

### 2.7. Statistical Analysis

The statistical analysis followed the 2 × 2 factorial design, with diet and quercetin as the main factors. No formal a priori sample-size calculation was performed for this experiment. The diet × quercetin interaction tested whether the association with quercetin differed according to dietary condition. Prespecified contrasts were HFD versus Control, HFD + quercetin versus HFD, and Control + quercetin versus Control. Given the modest and unequal final group sizes resulting from the two losses during follow-up described above, statistical inference emphasized effect estimates and their uncertainty and incorporated complementary robustness and sensitivity procedures, as detailed below. These procedures were used to characterize the stability of the findings and were not considered substitutes for additional biological replication.

Longitudinal phenotypic variables were analyzed using repeated-measures models that included diet, quercetin, time, and their interactions. An autoregressive first-order correlation structure was used when supported by model convergence. OGTT-derived AUC and iAUC were analyzed across baseline, week 8, and week 16. Week-16 AUC and iAUC were additionally evaluated by ANCOVA using the corresponding baseline measurement as covariate, and the complete week-16 glucose response was modeled across the five sampling times.

Terminal phenotypic outcomes and individual qPCR measurements were evaluated using factorial linear models. Heteroscedasticity-consistent HC3 standard errors were used for linear-model inference because final group sizes were modest and unequal. Multiplicity across the 21-gene panel was controlled using the Benjamini–Hochberg false-discovery-rate procedure; adjusted probabilities are reported as q values.

To characterize transcription beyond individual targets, the 21-gene matrix was standardized gene-wise and analyzed by unsupervised principal component analysis (PCA). Global group differences were assessed by PERMANOVA on Euclidean distances with 9999 permutations. Because the factorial design was unbalanced, sequential models were evaluated with both factor orders and the diet × quercetin component was also examined in a marginal framework. Homogeneity of multivariate dispersion was tested for the four-group factor and the two main factors.

Three predefined transcriptional modules were analyzed: m6A-related regulation, leptin–insulin-associated transcription, and melanocortin-associated transcription. Within each module, the first principal component (PC1) was used as a composite score for the dominant axis of transcriptional covariation. Gene standardization, PCA, and loadings were recalculated within stratified bootstrap resampling (10,000 iterations), and leave-one-sample-out analyses were used to assess stability.

Treatment-adjusted gene–gene covariance was evaluated for all 210 possible transcript pairs after centering expression within experimental group. Pearson correlations were complemented by rank-based sensitivity analysis, FDR correction, leave-one-sample-out assessment, and 10,000-iteration bootstrap resampling. As an additional small-sample regularization sensitivity analysis, Ledoit–Wolf shrinkage covariance estimation was applied to the same treatment-adjusted expression matrix. For the strongest robust cross-family associations, a group-stratified bootstrap (2000 iterations) preserved the four-group sample sizes and recalculated within-group centering and shrinkage at each iteration. Network edges represent residual co-variation and were not interpreted as evidence of regulatory direction, direct molecular interaction, or causality. All statistical analyses, including the complementary sensitivity analyses, were performed in R version 4.6.1 (R Foundation for Statistical Computing, Vienna, Austria). All tests were two-sided; q < 0.05 was used where multiplicity correction applied.

## 3. Results

### 3.1. Longitudinal Body Weight and Fasting Glucose

Body weight increased throughout the 16-week intervention in all four groups and showed the expected deceleration over time. A quadratic week term was strongly supported (F = 319.35, *p* < 0.001), whereas the diet × quercetin interaction did not materially modify the longitudinal weight trajectory. Absolute and percentage weight gain likewise showed substantial overlap among groups (Figure 2A–C).

Fasting glucose displayed a different longitudinal pattern. The diet × time component was significant after multiplicity adjustment (q < 0.001), whereas the three-way diet × quercetin × time interaction was not (q = 0.448). At week 16, fasting glucose was 27.22 mg/dL higher in HFD than in Control animals (q < 0.001). HFD + quercetin showed a mean difference of −6.10 mg/dL relative to HFD alone, which did not remain significant after adjustment (q = 0.222) (Figure 2D).

Food and energy intake were additionally summarized from the retained weekly group means (Supplementary Table S5). Across weeks 0–16, mean food-intake values averaged approximately 23.81, 23.68, 21.35, and 20.79 g for Control, Control + quercetin, HFD, and HFD + quercetin, respectively, whereas calculated energy-intake values averaged approximately 80.04, 79.55, 109.73, and 107.00 kcal. Thus, the HFD-fed groups consumed less food by mass but a higher calculated energy load than the control-fed groups, while Control and Control + quercetin and, separately, HFD and HFD + quercetin showed broadly similar descriptive intake trajectories. Energy intake during weeks 0–1 included the caloric contribution of the 10% fructose drinking solution in the HFD groups.

### 3.2. Glucose Tolerance and Terminal Metabolic Phenotype

OGTT responses diverged progressively during the intervention (Figure 3A–C). At week 8, baseline-adjusted AUC was 3201 mg·min/dL higher in HFD than in Control animals (q = 0.0023); the HFD + quercetin versus HFD contrast was not significant at this time point (q = 0.237). By week 16, the HFD–Control difference had increased to 6115 mg·min/dL (95% CI, 4884 to 7345; q < 0.001). In HFD-fed animals, quercetin was associated with an AUC reduction of 2415 mg·min/dL (95% CI, −3819 to −1011; q = 0.0029), and the diet × quercetin interaction for week-16 AUC was −2483 mg·min/dL (q = 0.0126). Control + quercetin and Control showed closely similar week-16 AUC values, supporting a diet-conditioned rather than uniform quercetin effect on this endpoint.

The incremental response showed a compatible pattern. Week-16 iAUC was 2571 mg·min/dL higher in HFD than in Control animals (95% CI, 1360 to 3781; q < 0.001), whereas HFD + quercetin showed an iAUC reduction of 1320 mg·min/dL relative to HFD (95% CI, −2511 to −130; q = 0.047). Longitudinal AUC trajectories are shown in Figure 3D.

Terminal adiposity and triglycerides provided the clearest non-glycemic metabolic contrasts (Figure 3E–H; Table 1). HFD increased retroperitoneal (visceral) fat by 8.27 g relative to Control (q < 0.001) and by 1.79 percentage points when expressed relative to body weight (q < 0.001). HFD + quercetin was lower than HFD by 5.16 g (95% CI, −7.37 to −3.05; q = 0.004) and 1.08 percentage points (95% CI, −1.59 to −0.57; q = 0.006), respectively. Triglycerides were 69.39 mg/dL higher in HFD than in Control (q < 0.001) and 47.03 mg/dL lower in HFD + quercetin than in HFD (95% CI, −60.65 to −34.34; q < 0.001). Total cholesterol was lower in HFD + quercetin than in HFD by 13.01 mg/dL, but this contrast did not remain significant after FDR correction (q = 0.118). No HFD + quercetin versus HFD differences were detected for HDL-C, LDL-C, brain weight, hypothalamus weight, or final body weight.

### 3.3. Global Hypothalamic Transcriptional Structure

Analysis of the complete 21-gene panel revealed a marked separation in global hypothalamic transcriptional structure among the experimental groups (Figure 4). PC1 accounted for 52.9% of standardized expression variance and PC2 for 20.6%, capturing 73.5% of total variance together. HFD samples were displaced along PC1, whereas HFD + quercetin occupied a region distinct from HFD and closer to the non-HFD groups.

A four-group PERMANOVA confirmed differences in multivariate transcriptional structure (R^2^ = 0.628, *p* = 0.0001). Multivariate dispersion did not differ significantly across the four groups (*p* = 0.0614), supporting interpretation of the omnibus group effect. In the factorial model, the diet × quercetin component accounted for 28.7% of multivariate variation (R^2^ = 0.287, *p* = 0.0001). Dispersion differed when samples were grouped by diet alone (*p* = 0.0002); the isolated main diet effect was therefore interpreted cautiously.

The heatmap provided a complementary representation of this structure. HFD animals showed coordinated differences across m6A-regulatory, leptin–insulin, melanocortin-associated, and oxidative-response transcripts, while HFD + quercetin displayed a distinct cross-domain expression pattern (Figure 4B).

### 3.4. Gene-Level Hypothalamic Transcriptional Response

Gene-level analysis showed that the global separation was accompanied by broad transcript-specific differences (Table 2). Fifteen of 21 transcripts met the predefined robustness criteria for the diet × quercetin interaction, and 16 of 21 did so for the HFD + quercetin versus HFD contrast. Robustness classification incorporated HC3 inference, Benjamini–Hochberg correction, 10,000-iteration stratified bootstrap resampling, and leave-one-sample-out sensitivity analysis. The Control + quercetin versus Control contrast was generally not FDR-significant across the panel, with FTO and NPY as the two exceptions; therefore, significant simple contrasts under HFD were not interpreted automatically as evidence of HFD-specificity.

Within the m6A-related group, HFD + quercetin differed most strongly from HFD for FTO, which was lower by 4.39 log2 units (95% bootstrap CI, −4.78 to −4.04; q < 0.001). METTL14 (+3.64 [3.01, 4.21]), METTL3 (+1.78 [1.18, 2.21]), RBM15B (+1.66 [1.23, 2.06]), and WTAP (+1.73 [0.68, 2.89]) were higher, with q < 0.001 for the first three and q = 0.031 for WTAP. ALKBH5, RBM15, VIRMA, YTHDC1, and ZC3H13 did not show robust HFD + quercetin versus HFD differences.

Technical quality-control data supported the magnitude of the FTO result (Supplementary Table S6; Figure S4). FTO and ACTB were available in technical triplicate for all 24 biological samples; median within-sample SDs were 0.062 and 0.061 cycles, respectively. The FTO standard curve had a slope of −3.411, amplification efficiency of 96.4%, and R^2^ = 0.9983, while ACTB showed a slope of −3.356, efficiency of 98.6%, and R^2^ = 0.9987. Mean melting temperatures were 81.65 °C for FTO and 84.21 °C for ACTB, and no Cq was recorded for the corresponding no-template controls. In the principal HFD comparison, FTO Ct values ranged from 24.19 to 30.13 in HFD and from 29.06 to 31.86 in HFD + quercetin animals. In the low-input series, FTO was detected in all eight replicates through the 0.03125 relative-input level, whereas detection declined at lower inputs. Leave-one-sample-out estimates for the HFD + quercetin versus HFD FTO contrast remained between −4.49 and −4.24 log2 units, indicating that the effect was not driven by a single biological sample.

A comparably broad response occurred among metabolic-signaling transcripts. Relative to HFD, HFD + quercetin showed higher LEPR (+1.77 log2 units), IRS1 (+1.39), PIK3R1 (+1.86), and AKT2 (+0.75), all with q < 0.001. FOXO1 (−1.38) and SOCS3 (−0.91) were lower (both q < 0.001). POMC showed one of the largest positive contrasts (+3.13 [2.23, 3.79], q < 0.001); AGRP (+1.84, q < 0.001), MC4R (+1.55, q = 0.002), NPY (+0.29, q = 0.023), and SOD2 (+1.07, q < 0.001) were also higher in HFD + quercetin than in HFD.

### 3.5. Coordinated Module-Level Transcriptional Response

To determine whether gene-level effects extended to coordinated transcriptional patterns, three predefined molecular modules were analyzed separately (Figure 5A–C). PC1 accounted for 47.0% of variance within the m6A-related module, 69.9% within the leptin–insulin-associated module, and 73.1% within the melanocortin-associated module.

**Figure 5:**
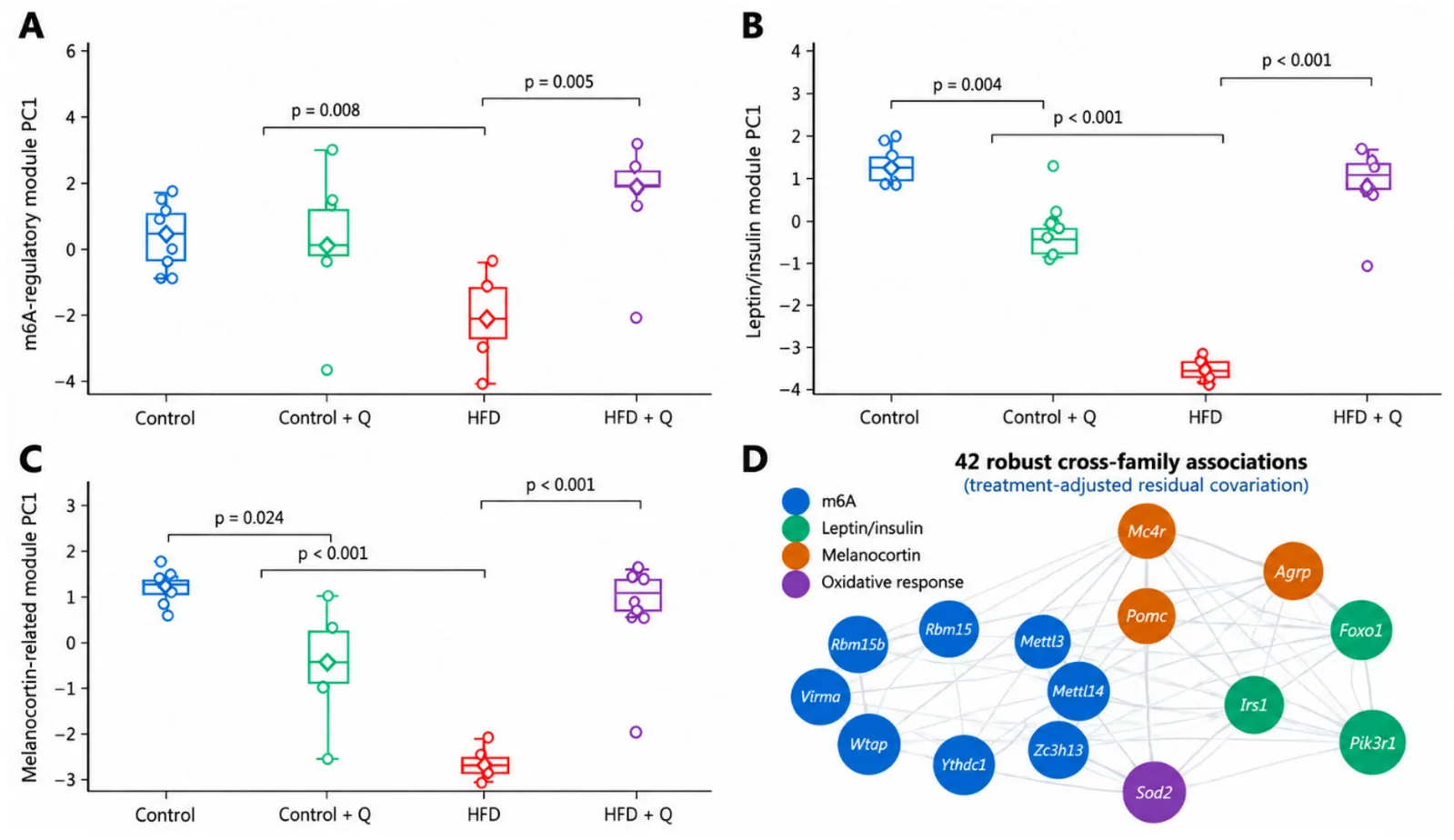
Coordinated hypothalamic transcriptional response and robust cross-family covariation. (**A**) m6A-related module PC1. (**B**) Leptin–insulin-associated module PC1. (**C**) Melanocortin-associated module PC1. Circles represent individual observations, whereas diamonds indicate group means; group distributions are also shown. (**D**) Network of 42 robust treatment-adjusted cross-family associations. Nodes are grouped by molecular domain; edges denote associations that remained robust across Pearson, rank-based, FDR, leave-one-sample-out, and bootstrap analyses. Network edges are undirected and do not imply causal regulation. Additional shrinkage sensitivity is reported in Table 3 and Supplementary Table S7.

**Table 3:** Multilevel robustness of the hypothalamic molecular findings.

Level	Endpoint	Test	Estimate	*p*/q	Note
Global molecular profile	PC1 + PC2	Variance explained	73.5%		PC1 52.9%; PC2 20.6%
Global molecular profile	Four experimental groups	PERMANOVA omnibus	R^2^ = 0.628	<0.001	Dispersion *p* = 0.061
Global molecular profile	Diet × Quercetin	PERMANOVA component	R^2^ = 0.287	<0.001	Diet-only dispersion *p* = <0.001; interpret diet main effect cautiously.
Nested module	m6A-regulatory	Diet × Quercetin	4.33 [1.55, 6.84]	0.028	ROBUST
Nested module	Leptin–insulin-associated	Diet × Quercetin	5.97 [4.69, 6.92]	<0.001	ROBUST
Nested module	Melanocortin-related	Diet × Quercetin	4.90 [3.44, 6.19]	<0.001	ROBUST
Nested module	m6A-regulatory	HFD + Q vs. HFD	3.89 [0.97, 5.89]	0.005	ROBUST
Nested module	Leptin–insulin-associated	HFD + Q vs. HFD	4.59 [3.52, 5.25]	<0.001	ROBUST
Nested module	Melanocortin-related	HFD + Q vs. HFD	3.22 [2.18, 4.09]	<0.001	ROBUST
Network	All adjusted gene–gene associations	Sensitivity classification	70/210 ROBUST		Adjusted Pearson + rank sensitivity + bootstrap + LOO
Network	Cross-family associations	ROBUST residual co-variation	42 edges		Undirected associations; no causal interpretation
Network	All adjusted associations	Ledoit–Wolf shrinkage sensitivity	54/70 robust retain |r| ≥ 0.50	—	λ = 0.225; 210/210 directions preserved; |r| rank ρ = 0.963
Network	Cross-family robust associations	Shrinkage + stratified bootstrap	31/42 retain |r| ≥ 0.50	—	Residualization and shrinkage recalculated within 2000 group-stratified resamples

Module estimates correspond to first-principal-component transcriptional scores. Confidence intervals derive from nested bootstrap analyses in which gene standardization, PCA, and component loadings were recalculated within each iteration. Ledoit–Wolf results provide a regularized sensitivity analysis of the treatment-adjusted covariance structure and do not imply causal regulation. Q, quercetin; HFD, high-fat diet; PCA, principal component analysis; PERMANOVA, permutational multivariate analysis of variance. In this table, ‘robust’ indicates that the finding met the predefined statistical sensitivity criteria; it does not imply independent experimental replication, mechanistic validation, or biological confirmation.

All three modules showed evidence of a diet × quercetin interaction that remained robust under nested sensitivity analysis. Interaction estimates were 4.33 (95% bootstrap interval, 1.55 to 6.84; q = 0.028) for the m6A-related module, 5.97 (4.69 to 6.92; q < 0.001) for the leptin–insulin-associated module, and 4.90 (3.44 to 6.19; q < 0.001) for the melanocortin-associated module. The HFD + quercetin versus HFD contrasts were 3.89 (0.97 to 5.89; q = 0.005), 4.59 (3.52 to 5.25; q < 0.001), and 3.22 (2.18 to 4.09; q < 0.001), respectively. Module scores summarize dominant transcriptional covariation and are not measures of pathway activation.

### 3.6. Robust Cross-Family Transcriptional Covariation

Treatment-adjusted covariance was then evaluated across the 210 possible transcript pairs. Seventy associations met the predefined criteria for robust covariation, while 46 were classified as moderate, and 94 as unstable. Forty-two robust associations connected transcripts assigned to different molecular families (Figure 5D). Ledoit–Wolf shrinkage provided an additional regularization sensitivity analysis: the estimated shrinkage intensity was 0.225, the direction of all 210 residual associations was preserved, and the rank ordering of absolute association strength remained highly concordant with the original Pearson estimates (Spearman ρ = 0.963). Fifty-four of the 70 originally robust associations retained an absolute shrinkage correlation ≥ 0.50, including 31 of the 42 robust cross-family associations (Supplementary Table S7).

Among the strongest cross-family associations, AGRP and PIK3R1 showed an adjusted Pearson correlation of r = 0.91 (q < 0.001; bootstrap interval, 0.70 to 0.97). Strong residual covariation was also observed for METTL3-PIK3R1 (r = 0.87), METTL3-AGRP (r = 0.86), MC4R-SOD2 (r = 0.86), METTL3-MC4R (r = 0.84), and POMC-PIK3R1 (r = 0.82). The direction of these associations was retained in rank-based, leave-one-sample-out, resampling, and shrinkage analyses. For the strongest cross-family associations, group-stratified shrinkage bootstrap resampling recalculated treatment centering and regularization in each iteration, and residual-expression scatterplots are provided in Supplementary Figure S3. The covariance structure therefore represents reproducible residual co-variation after removal of experimental-group means; it does not establish regulatory direction, direct molecular interaction, or causality.

## 4. Discussion

The principal finding of this study is that quercetin exposure was associated with broad, diet-conditioned reorganization of hypothalamic transcription rather than isolated shifts in a small number of metabolic genes. Sixteen of the 21 transcripts differed robustly between HFD + quercetin and HFD animals, and 15 of 21 showed a robust diet × quercetin interaction, spanning m6A-related, leptin–insulin-associated, melanocortin-associated, and oxidative-response domains. This gene-level pattern was supported by the global PCA/PERMANOVA structure, by robust differences in all three predefined transcriptional modules, and by cross-family residual covariance. The molecular response occurred alongside improved glucose tolerance, lower retroperitoneal adiposity, and lower triglycerides without a major difference in final body weight. Prior experimental work has independently linked quercetin to metabolic improvement and hypothalamic responses, while HFD-associated FTO biology provides a mechanistic context linking m6A-related regulation and leptin responsiveness [12,13,14,15].

The dissociation between final body weight and metabolic improvement is biologically informative. Quercetin-treated HFD animals had lower visceral adiposity, triglycerides, and OGTT responses even though terminal body weight remained comparable with untreated HFD animals. Quercetin has produced similar patterns in rodent models, including reductions in adiposity, dyslipidemia, glucose intolerance, adipose inflammation, or insulin resistance without a uniform requirement for large weight loss [13,14,20,21,22,23]. Kábelová et al. also showed that metabolic improvement with quercetin can coexist with extensive transcriptional remodeling in metabolically active tissues, supporting the broader concept that quercetin-responsive phenotypes may reflect tissue-level regulatory changes rather than simple reduction in body mass [20].

The factorial structure makes the dietary context of the response explicit. Significant diet × quercetin interactions were observed for week-16 glucose AUC, retroperitoneal adiposity, triglycerides, and a broad subset of hypothalamic transcripts. Control + quercetin and Control were broadly similar for the major metabolic outcomes, whereas HFD + quercetin differed substantially from HFD for glucose tolerance, adiposity, and triglycerides. This context dependence was not universal at the transcript level: FTO and NPY also showed FDR-significant Control + quercetin versus Control differences. The findings therefore support a predominantly HFD-context-dependent metabolic response to quercetin rather than an effect that is strictly restricted to HFD exposure. A plausible explanation is that the HFD state creates metabolic and inflammatory dysregulation with greater scope for quercetin-responsive remodeling, whereas control-fed animals are closer to homeostatic baseline; this interpretation remains a hypothesis for direct mechanistic testing.

The hypothalamic response extends this metabolic phenotype into a central tissue that integrates nutrient and endocrine information. Quercetin has previously reduced HFD-associated hypothalamic inflammatory cytokines and microglial activation [15]. Orally administered quercetin and methylated derivatives have also been detected in perfused rat brain tissue, demonstrating that quercetin-derived compounds can reach the central nervous system under experimental conditions [24]. The present data do not resolve whether the transcriptional response originated from direct exposure of hypothalamic cells, circulating metabolites, or systemic signals generated by improved peripheral metabolism. They establish that the hypothalamic transcriptional state is a component of the response observed in the HFD + quercetin condition [15,24].

Within the molecular panel, the marked reduction in FTO expression in HFD + quercetin animals deserves specific consideration. Manipulation of hypothalamic FTO has long been known to influence feeding behavior, and more recent work has connected hypothalamic FTO to leptin responsiveness and HFD-induced metabolic dysfunction [12,21,22]. Inhibition of hypothalamic FTO can enhance ERK1/2-STAT3 signaling and reduce food intake and body weight, while selective loss of FTO in leptin-receptor-expressing neurons partially attenuates HFD-induced leptin resistance [12,25]. The pronounced FTO contrast therefore identifies a plausible transcriptional point of convergence between nutritional exposure, m6A-related regulation, and leptin biology. It should not be interpreted as evidence that global hypothalamic m6A abundance increased, because expression of a demethylase does not determine transcript-specific methylation stoichiometry or reader engagement [12,25,26]. FTO was also lower in Control + quercetin than in Control, while the diet × quercetin interaction for FTO was not significant after FDR correction. Thus, although FTO expression was markedly lower in HFD + quercetin than in HFD animals, this contrast does not demonstrate that the effect of quercetin on FTO differed according to dietary condition. FTO is therefore better interpreted as a quercetin-associated transcriptional response rather than as an HFD-specific effect.

The accompanying METTL3 and METTL14 responses place the current results within a rapidly developing area of hypothalamic epitranscriptomics. Li et al. demonstrated that POMC-neuron METTL14, YTHDC1, and YTHDF2 are required for normal energy balance and identified an m6A-dependent METTL14/YTHDC1/POMC pathway; METTL14 overexpression in POMC neurons protected against diet-induced obesity [27]. Shen et al. independently showed that loss of METTL14 during hypothalamic development impairs generation of feeding-related neurons and produces adult obesity and altered glucose-insulin homeostasis, with related phenotypes after disruption of METTL3 or YTHDC1 [24]. The concurrent increase in METTL14, METTL3, and POMC in HFD + quercetin animals is therefore mechanistically interesting. Our experiment does not establish that quercetin altered methylation of POMC or another specific RNA, but it identifies a coordinated transcriptional configuration that is directly testable with transcript-specific m6A methods [27,28].

Tissue context is essential when interpreting m6A-regulatory genes. METTL14 is not uniformly metabolically beneficial across organs. Adipocyte METTL14 can suppress beta-adrenergic signaling and lipolysis, promoting HFD-associated obesity and insulin resistance, whereas hepatic METTL14 can increase m6A-dependent G6pc biosynthesis and hepatic glucose production [29,30]. These effects differ from the protective role reported in hypothalamic POMC neurons [27,28]. The contrast illustrates why the present whole-hypothalamus data should be interpreted as transcriptional remodeling of m6A-associated machinery rather than a global change in m6A activity with a predetermined metabolic direction [27,28,29,30].

The leptin–insulin-associated component provides a second coherent layer of the transcriptional response. HFD + quercetin animals showed higher LEPR, IRS1, PIK3R1, and AKT2 expression together with lower SOCS3 and FOXO1 relative to HFD. Region-specific leptin resistance is a characteristic feature of diet-induced obesity, and SOCS3 contributes directly to this process: neural Socs3 deficiency enhances leptin sensitivity, whereas increased SOCS3 within AgRP neurons can participate in the early development of HFD-associated cellular leptin resistance [31,32,33]. Hypothalamic FTO has also been linked to SOCS3 and impaired leptin-STAT3 signaling under HFD conditions [12]. The lower SOCS3 and higher LEPR expression observed with quercetin therefore fit a biologically coherent transcriptional context, while remaining distinct from direct evidence of improved receptor signaling [12,31,32,33].

The IRS-PIK3R1-AKT-FOXO1 pattern is similarly compatible with altered central metabolic regulation, but transcript abundance and signaling activity must remain conceptually separate. Chronic HFD can impair leptin responsiveness and change the electrophysiological behavior of POMC neurons [34]. PIK3R1 signaling is required for key acute actions of leptin in POMC neurons and contributes to hypothalamic control of energy expenditure [35,36]. FOXO1 integrates insulin- and leptin-related signals and regulates appetite-associated transcription; experimental manipulation of hypothalamic or POMC-neuron FOXO1 changes food intake, body weight, and neuropeptide regulation [37,38,39]. Thus, higher IRS1, PIK3R1, and AKT2 together with lower FOXO1 define a transcriptional state compatible with altered leptin–insulin-associated regulation, but circulating leptin and insulin concentrations, receptor activation, and phosphorylation of AKT, FOXO1, STAT3, or the leptin receptor were not measured [30,31,32,33,34,35]. Consequently, the present data do not directly establish central or systemic leptin/insulin resistance or its reversal.

The melanocortin-associated findings argue against reducing the response to a simple anorexigenic-versus-orexigenic switch. POMC and MC4R were substantially higher in HFD + quercetin than in HFD, yet AGRP and NPY also increased. Dietary composition, inflammatory state, and duration of HFD exposure can change the direction and functional meaning of hypothalamic neuropeptide responses [34,40,41]. Recent cell-specific work adds another layer: FTO in AgRP neurons regulates Kif1a splicing, axonal vesicle trafficking, and secretion of NPY/AgRP-containing dense-core vesicles, demonstrating that transcript abundance and neuropeptide output need not move in parallel [42]. The simultaneous increase in transcripts with opposing physiological functions may reflect compensatory responses, differences among hypothalamic neuronal populations, or broader remodeling of melanocortin-associated transcription. Because neuronal activity, neuropeptide release, and functional melanocortin signaling were not measured, the net physiological consequence of this expression pattern cannot be determined from the present data. The findings are therefore better described as melanocortin-associated transcriptional remodeling than as restoration of a fixed anorexigenic/orexigenic balance [34,40,41,42].

The multilevel analysis strengthens the interpretation that quercetin-associated molecular change was coordinated. The global expression structure, three predefined modules, and residual cross-family covariance converge on a distributed transcriptional response rather than dependence on FTO, POMC, or any single target. The additional Ledoit–Wolf analysis attenuated correlation magnitudes, as expected with shrinkage, but preserved the direction of all 210 residual associations and retained substantial magnitude for most originally robust edges; this supports structural stability without converting statistical co-variation into a causal network. Given the number of transcript-pair associations evaluated relative to the biological sample size, these estimates should be interpreted cautiously. Shrinkage regularization improves the stability of covariance estimates under these conditions, but it does not provide independent biological replication or experimental validation of the inferred associations. Experimental studies provide direct mechanistic links for selected systems represented within these domains, including FTO with leptin resistance, METTL14/YTHDC1 with POMC regulation, and FTO-dependent regulation of AgRP-neuron vesicle trafficking. The present covariance analysis does not reproduce those mechanisms directly; it maps treatment-adjusted transcriptional co-variation that can be used to prioritize mechanistic experiments [12,27,28,42].

The origin of the hypothalamic transcriptional response may involve both central and systemic routes. Brain exposure to quercetin-derived compounds has been demonstrated experimentally [20], but peripheral mechanisms can also generate signals capable of changing central metabolic state. In mice, quercetin reshaped gut microbiota and bile-acid composition, increasing non-12-hydroxylated bile acids and TGR5-dependent thermogenic responses; fecal microbiota transfer reproduced part of that metabolic phenotype [43]. A separate 2025 study linked quercetin-driven Akkermansia muciniphila enrichment to indole-3-lactic acid and an FTO/m6A/YTHDF2/CYP8B1 pathway controlling bile-acid metabolism [44]. These mechanisms were demonstrated outside the hypothalamus and should not be transferred directly to our tissue. They do support a physiologic model in which hypothalamic transcriptional remodeling can coexist with, and potentially respond to, quercetin-induced peripheral metabolic communication [24,33,44].

Several features of the experimental design limit the scope of inference. Final group sizes were small and unequal (Control, n = 8; Control + quercetin, n = 5; HFD, n = 5; HFD + quercetin, n = 6), which limits statistical precision, particularly for interaction effects. HC3 inference, FDR correction, stratified bootstrap resampling, leave-one-sample-out assessment, multivariate-dispersion testing, and shrinkage covariance sensitivity were used to examine result stability, but these approaches do not replace independent biological replication. The dietary model also warrants cautious interpretation. Although the experimental groups are referred to as HFD for consistency, the experimental diet differed from the control diet not only in fat content but also in carbohydrate composition, protein contribution, and energy density. In addition, the HFD groups received 10% fructose in the drinking fluid during weeks 0–1. The observed metabolic and transcriptional differences therefore cannot be attributed exclusively to dietary fat and should be interpreted in the context of the overall high-fat/high-carbohydrate experimental model. RNA was obtained from whole hypothalamic tissue, so these data cannot identify which neuronal or glial populations contributed to the observed transcriptional differences. Cell-specific studies of METTL14, YTHDC1, FTO, and POMC/AgRP neurons illustrate the additional resolution that targeted approaches can provide [23,24,30,38]. The m6A-related findings reflect expression of genes involved in regulatory machinery rather than direct measurements of global or transcript-specific RNA methylation. Protein-level validation was not performed as part of the present transcriptional study; changes in transcript abundance therefore do not establish corresponding changes in protein abundance or phosphorylation-dependent signaling. Circulating leptin and insulin were also not measured. We therefore interpret the leptin–insulin and melanocortin findings as associated transcriptional profiles and the covariance structure as undirected transcriptional association rather than evidence of pathway activation or causal regulation. Only male rats were studied, and whether the same responses occur in females remains unknown.

The design also provides complementary strengths. Metabolic status was followed longitudinally, food/fluid and energy intake were monitored descriptively, OGTTs were repeated across the intervention, terminal adiposity and lipids were measured within the same factorial framework, and the molecular analysis covered 21 hypothalamic targets rather than a single candidate gene. Molecular findings were evaluated at four levels: individual transcripts, global multivariate structure, predefined biological modules, and treatment-adjusted covariance. This approach extends prior work focused primarily on peripheral metabolic outcomes, hypothalamic inflammation, or individual m6A regulators [13,14,15,16,23,24]. The use of resampling, influence analyses, and shrinkage regularization adds information about result stability that is particularly useful for a targeted molecular panel with unequal final group sizes [16,23,24].

The combined metabolic and molecular data support a model in which quercetin attenuates selected consequences of HFD feeding while reshaping the hypothalamic transcriptional state. FTO, METTL3, METTL14, LEPR, SOCS3, FOXO1, POMC, and the cross-domain covariance structure emerge as priority targets because independent experimental studies connect these systems to leptin responsiveness, energy homeostasis, and cell-specific m6A regulation [12,23,24,38]. Peripheral microbiota-bile-acid and m6A-linked mechanisms may contribute to the systemic signals surrounding this central response [39,40]. Direct m6A mapping, protein-level measurements, functional signaling assays, and cell-type-resolved analyses will be required to determine which components are causal, but the present data define a reproducible transcriptional architecture for that next stage of investigation [12,23,24,38,39,40].

The relevance of these findings to human metabolic health should be considered in the context of heterogeneous clinical responses to quercetin supplementation. Human randomized trials have not consistently demonstrated reductions in body weight or fasting glucose, although some studies and subgroup analyses have reported improvements in selected cardiometabolic outcomes [41,42,43]. This pattern is compatible with the present observation that quercetin improved glucose tolerance, visceral adiposity, and triglycerides without significantly reducing final body weight. Rather than supporting a simple weight-loss effect, these findings raise the possibility that metabolic responses to quercetin may involve tissue-specific regulatory adaptations that are not necessarily reflected by changes in body mass. The hypothalamus is particularly relevant to this interpretation because it integrates circulating nutritional and hormonal signals and coordinates energy and glucose homeostasis.

Nevertheless, extrapolation of the present molecular findings to humans requires caution. The current study was performed in male Wistar rats receiving quercetin at 100 mg/kg/day, an experimental efficacy dose that is substantially different from usual dietary exposure in humans, and the hypothalamic analyses were based on transcriptional abundance rather than direct measurements of RNA methylation, protein abundance, enzymatic activity, or signaling-pathway activation. Accordingly, the observed changes in Fto, Mettl3, Mettl14, Lepr, Socs3, Foxo1, Pomc, and related transcripts should be considered mechanistic candidates rather than validated human targets. Direct dose equivalence to normal human dietary quercetin intake is not implied. Human translation will require pharmacokinetic and bioavailability studies at clinically relevant exposures, metabolic phenotyping, and, where feasible, molecular biomarkers of m6A-related regulation and leptin–melanocortin-associated biology.

From a nutritional implementation perspective, the present findings should therefore be viewed as a mechanistic framework rather than evidence of clinical efficacy. The identified transcriptional architecture provides experimentally testable hypotheses for future nutritional intervention studies, including whether variation in m6A-related regulation and leptin–melanocortin-associated transcription is associated with differences in metabolic response to quercetin. Such an approach may be particularly informative for moving beyond anthropometric outcomes and toward mechanistically informed nutritional strategies.

## 5. Conclusions

Quercetin attenuated selected metabolic alterations associated with HFD feeding, particularly impaired glucose tolerance, retroperitoneal adiposity, and hypertriglyceridemia, without producing a major difference in final body weight. The strongest metabolic effects were diet-conditioned and predominantly evident under HFD exposure. This response was accompanied by broad changes in hypothalamic gene expression involving m6A-related transcripts and genes associated with leptin–insulin and melanocortin biology. The same molecular signal was retained across individual-gene, multivariate, module-level, covariance, and shrinkage-sensitivity analyses.

The pronounced responses of Fto, Mettl3, Mettl14, Lepr, Socs3, Foxo1, and Pomc identify molecular targets for mechanistic follow-up. The findings support hypothalamic transcriptional remodeling as a component of the quercetin response, but they do not constitute direct evidence of altered m6A methylation, circulating leptin or insulin, protein abundance, or signaling-pathway activation. Cell-type-resolved experiments, transcript-specific m6A measurements, and protein-level and functional signaling analyses are therefore required to establish causal relationships among these molecular systems.

From a translational perspective, these findings identify candidate molecular mechanisms for further investigation rather than establishing hypothalamic m6A-dependent mediation of quercetin’s metabolic effects. The study was conducted exclusively in male rats receiving quercetin at 100 mg/kg/day, an experimental dose that does not represent usual human dietary exposure. Translation to human nutrition will therefore require confirmation of the molecular targets, direct assessment of m6A-related mechanisms and pathway activity, evaluation in both sexes, clinically relevant exposure levels, and well-controlled human intervention studies.

## Figures and Tables

**Figure 1:**
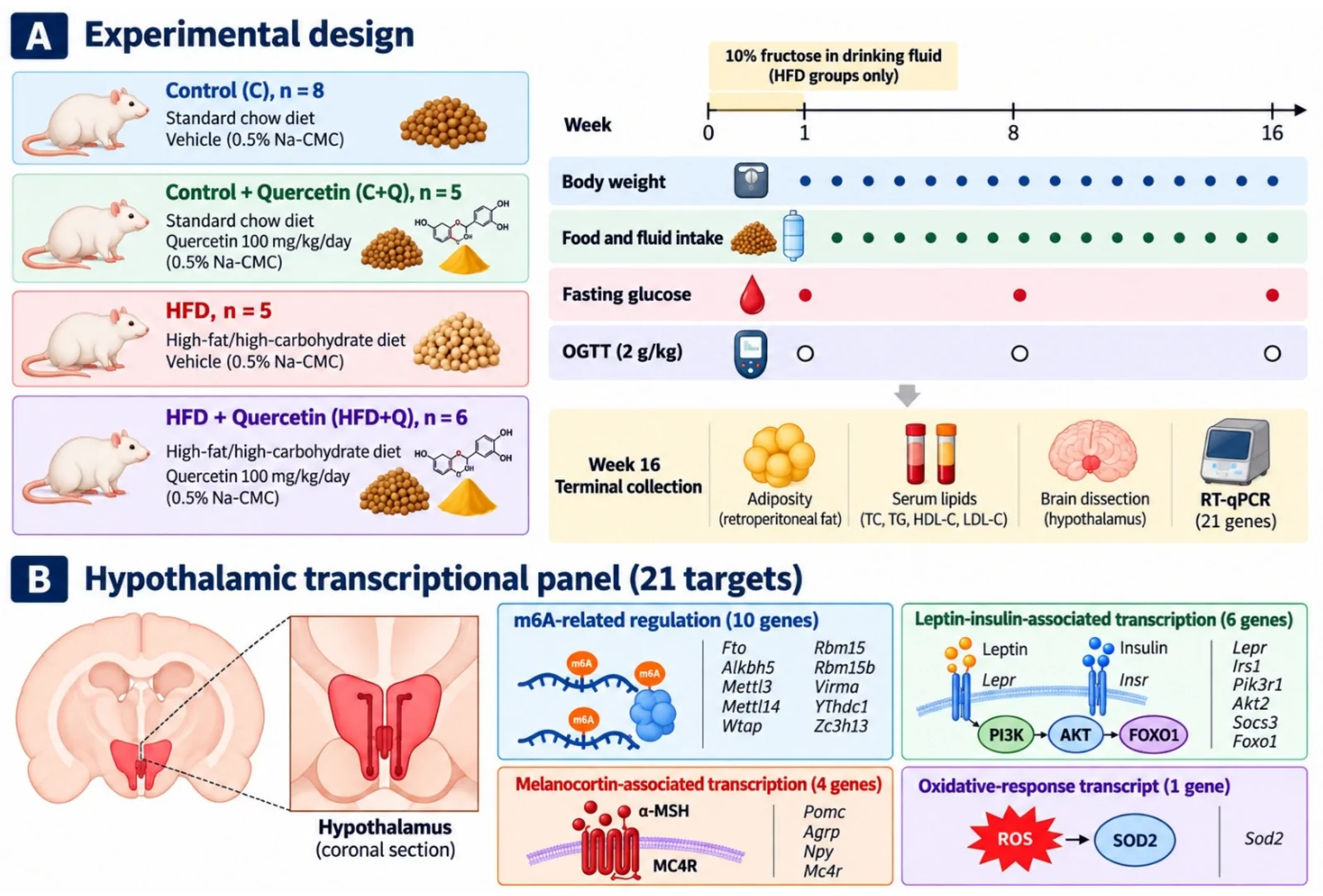
Experimental design and hypothalamic transcriptional panel. (**A**) Four experimental groups were followed for 16 weeks. HFD groups received 10% (*w*/*v*) fructose in the drinking fluid during weeks 0–1. Body weight, fasting glucose, and food/fluid intake were monitored longitudinally; OGTTs were performed at weeks 0, 8, and 16, followed by terminal metabolic and tissue collection. (**B**) RT-qPCR targeted 21 hypothalamic transcripts assigned to m6A-related, leptin–insulin-associated, melanocortin-associated, and oxidative-response domains. Molecular measurements represent relative mRNA abundance. HFD, high-fat diet; Q, quercetin.

**Figure 2:**
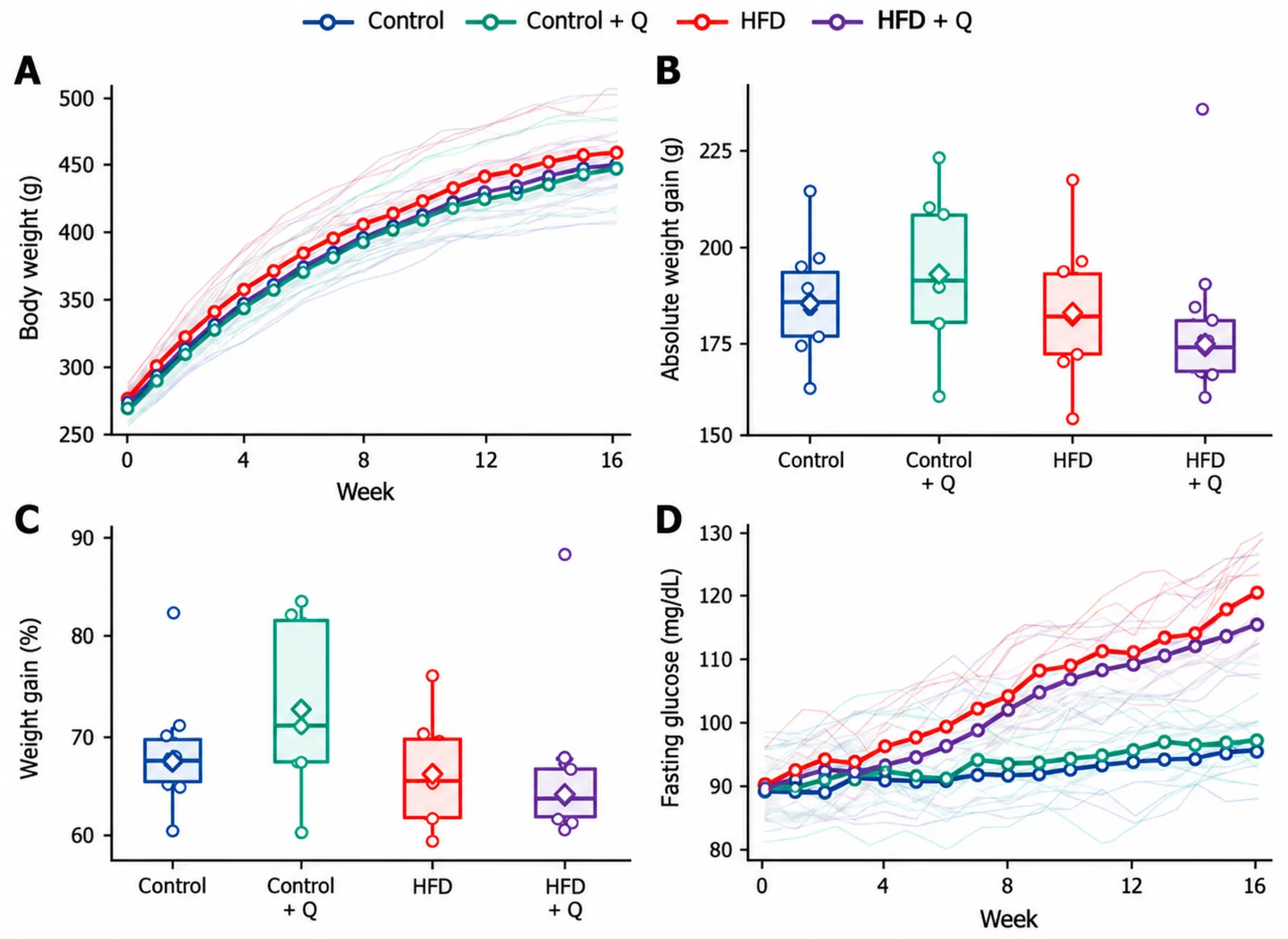
Longitudinal metabolic phenotype. (**A**) Body-weight trajectories across 16 weeks. (**B**) Absolute body-weight gain. (**C**) Percentage body-weight gain. (**D**) Longitudinal fasting glucose. Thin lines indicate individual trajectories and thicker lines indicate group means; distribution panels show individual observations and group summaries. Q, quercetin; HFD, high-fat diet.

**Figure 3:**
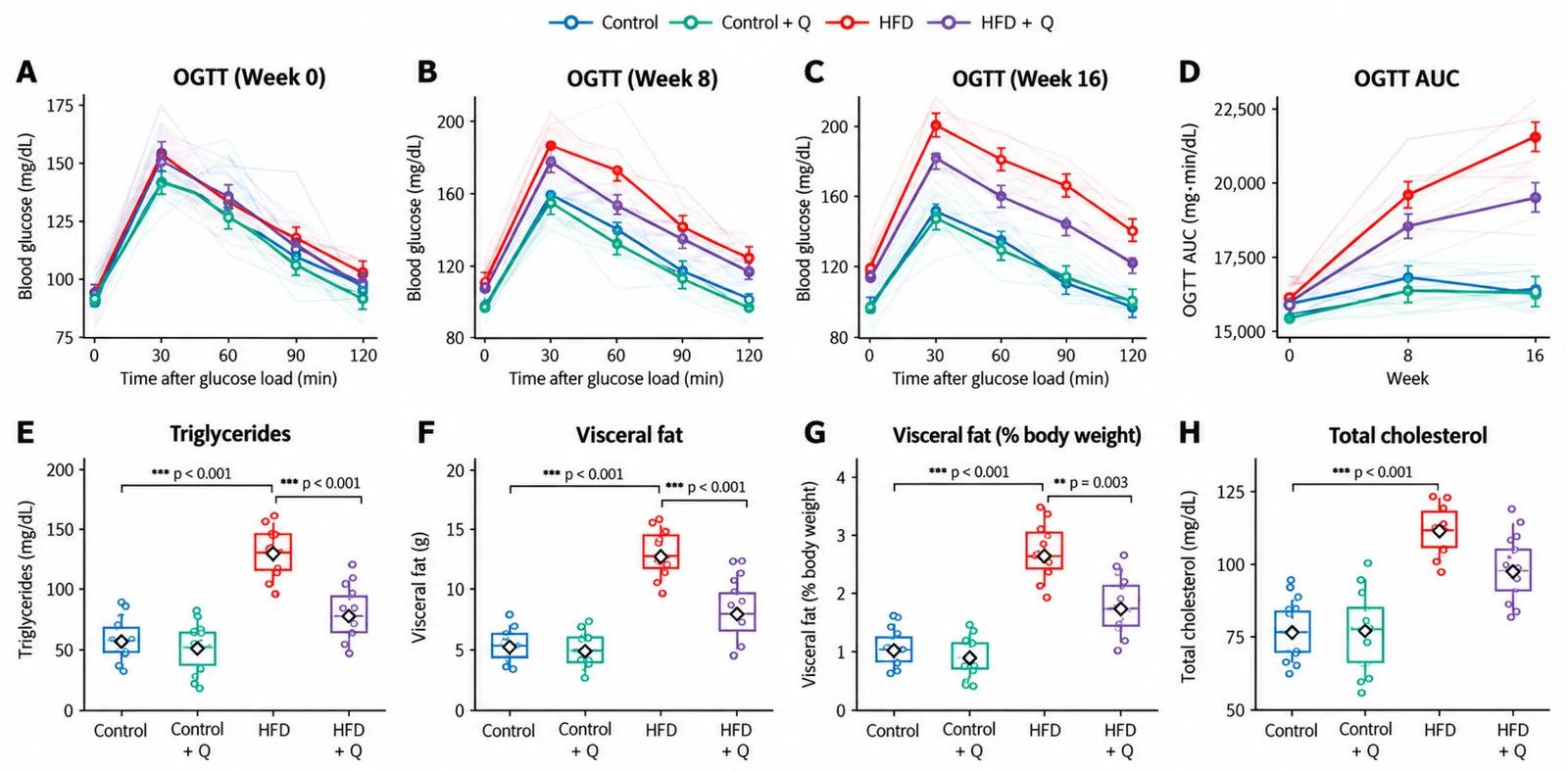
Glucose tolerance and terminal metabolic phenotype. (**A**–**C**) OGTT curves at weeks 0, 8, and 16, respectively. (**D**) Longitudinal OGTT AUC. (**E**–**H**) Triglycerides, retroperitoneal adipose mass used as the visceral-fat measure, retroperitoneal adipose mass expressed as percentage of body weight, and total cholesterol. Thin lines in panels A–D indicate individual animal trajectories, whereas thicker lines indicate group mean profiles. Asterisks above brackets indicate statistical significance for the prespecified model-based contrasts (** *p* < 0.01; *** *p* < 0.001); exact *p* values are shown in the figure, and FDR-adjusted q values are reported in Table 1.

**Figure 4:**
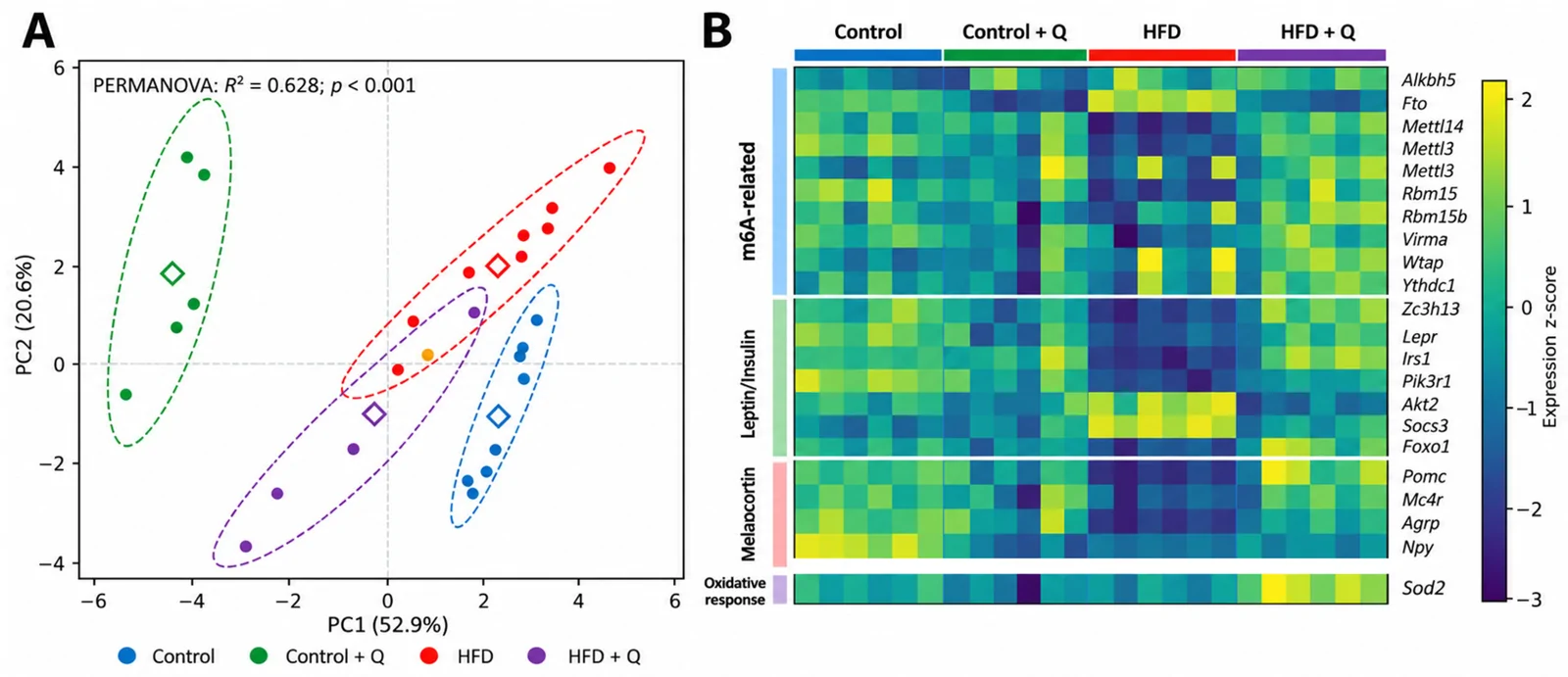
Global hypothalamic transcriptional profile. (**A**) Unsupervised PCA of standardized expression values for 21 hypothalamic transcripts. PC1 and PC2 accounted for 52.9% and 20.6% of total variance, respectively; diamonds denote group centroids and dashed ellipses provide a descriptive view of within-group spread. (**B**) Heatmap of gene-wise standardized expression values ordered by experimental group and molecular domain. PIK3R1 denotes the Pik3r1 assay (RefSeq NM_013005.3).

**Table 1:** Experimental phenotype and terminal metabolic outcomes.

Outcome	Unit	Control	Control + Q	HFD	HFD + Q	Diet × Q Effect [95% CI]	Diet × Q q	HFD + Q vs. HFD Effect [95% CI]	HFD + Q vs. HFD q
Brain weight	g	1.92 ± 0.07	1.93 ± 0.08	1.85 ± 0.10	1.89 ± 0.10	0.03 [−0.11, 0.16]	0.948	0.04 [−0.07, 0.14]	0.603
Fasting glucose	mg/dL	94.06 ± 6.32	95.52 ± 5.64	121.28 ± 7.81	115.18 ± 10.83	−7.55 [−18.73, 4.31]	0.412	−6.10 [−15.73, 3.87]	0.452
Final body weight	g	453.01 ± 11.58	454.94 ± 21.51	458.16 ± 26.14	450.75 ± 30.69	−9.34 [−44.68, 26.06]	0.679	−7.41 [−36.73, 23.43]	0.700
HDL cholesterol	mg/dL	45.94 ± 4.59	51.22 ± 5.35	35.72 ± 7.28	38.95 ± 5.95	−2.05 [−10.73, 6.94]	0.883	3.23 [−3.91, 10.53]	0.482
Hypothalamus weight	mg	41.34 ± 2.52	41.86 ± 1.80	39.70 ± 1.33	40.33 ± 1.87	0.11 [−2.67, 2.80]	0.948	0.63 [−1.09, 2.30]	0.603
LDL cholesterol	mg/dL	17.16 ± 11.74	11.99 ± 13.22	49.96 ± 13.68	43.13 ± 8.41	−1.66 [−19.35, 15.85]	0.883	−6.83 [−19.16, 5.13]	0.482
Total cholesterol	mg/dL	75.50 ± 12.28	73.16 ± 17.98	112.14 ± 10.11	99.13 ± 9.13	−10.67 [−29.78, 8.84]	0.771	−13.01 [−23.19, −2.67]	0.118
Triglycerides	mg/dL	62.91 ± 18.74	59.82 ± 20.60	132.30 ± 13.82	85.27 ± 9.96	−43.94 [−68.21, −20.09]	0.033	−47.03 [−60.65, −34.34]	<0.001
Retroperitoneal fat	g	5.80 ± 1.49	5.64 ± 1.20	14.07 ± 1.79	8.91 ± 2.23	−5.01 [−7.57, −2.53]	0.019	−5.16 [−7.37, −3.05]	0.004
Retroperitoneal fat	% body weight	1.28 ± 0.35	1.25 ± 0.32	3.08 ± 0.40	1.99 ± 0.56	−1.05 [−1.66, −0.45]	0.025	−1.08 [−1.59, −0.57]	0.006

Values are mean ± SD for descriptive group summaries. Effects are factorial-model estimates with HC3 inference; q values are Benjamini–Hochberg-adjusted where applicable. Q, quercetin; HFD, high-fat diet; HDL-C, high-density lipoprotein cholesterol; LDL-C, low-density lipoprotein cholesterol.

**Table 2:** Gene-level hypothalamic transcriptional effects.

Gene	Diet × Q Effect [95% CI]	Forest	q	HFD + Q vs. HFD Effect [95% CI]	Forest	q
Alkbh5	−0.81 [−1.57, 0.00]	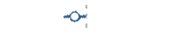	0.158	0.12 [−0.27, 0.50]	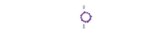	0.686
Fto	−0.36 [−0.80, 0.05]	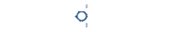	0.220	−4.39 [−4.78, −4.04]	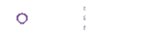	<0.001
Mettl14	3.44 [2.58, 4.29]	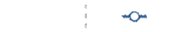	<0.001	3.64 [3.01, 4.21]	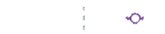	<0.001
Mettl3	2.52 [1.59, 3.37]	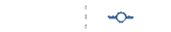	<0.001	1.78 [1.18, 2.21]	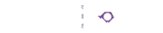	<0.001
Rbm15	−0.50 [−1.49, 0.42]	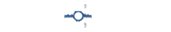	0.440	0.11 [−0.72, 0.86]	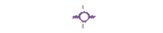	0.826
Rbm15b	1.96 [1.28, 2.62]	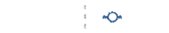	<0.001	1.66 [1.23, 2.06]	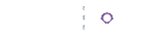	<0.001
Virma	0.84 [−0.13, 1.84]	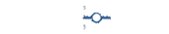	0.223	0.51 [−0.26, 1.27]	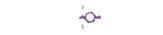	0.382
Wtap	2.41 [0.99, 3.96]	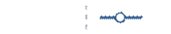	0.030	1.73 [0.68, 2.89]	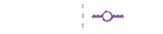	0.031
Ythdc1	0.15 [−0.72, 1.01]	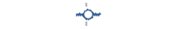	0.789	0.16 [−0.55, 0.81]	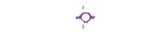	0.758
Zc3h13	0.57 [−0.46, 1.61]	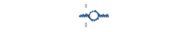	0.437	0.32 [−0.54, 1.10]	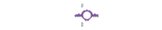	0.627
Akt2	1.32 [0.88, 1.72]	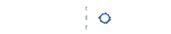	<0.001	0.75 [0.48, 1.00]	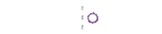	<0.001
Foxo1	−1.84 [−2.44, −1.22]	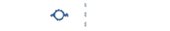	<0.001	−1.38 [−1.69, −1.14]	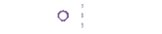	<0.001
Irs1	2.63 [1.77, 3.46]	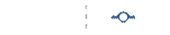	<0.001	1.39 [0.91, 1.80]	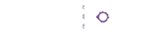	<0.001
Pik3r1	1.80 [1.08, 2.42]	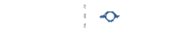	<0.001	1.86 [1.30, 2.24]	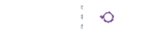	<0.001
Socs3	−0.79 [−1.11, −0.50]	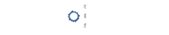	<0.001	−0.91 [−1.06, −0.75]	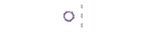	<0.001
Agrp	2.57 [1.57, 3.51]	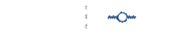	<0.001	1.84 [1.17, 2.37]	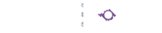	<0.001
Lepr	2.36 [1.55, 3.07]	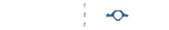	<0.001	1.77 [1.13, 2.21]	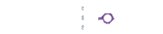	<0.001
Mc4r	2.22 [1.10, 3.42]	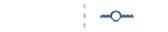	0.010	1.55 [0.79, 2.13]	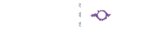	0.002
Npy	2.00 [1.69, 2.27]	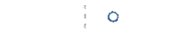	<0.001	0.29 [0.10, 0.44]	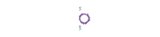	0.023
Pomc	3.36 [2.33, 4.23]	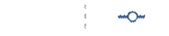	<0.001	3.13 [2.23, 3.79]	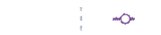	<0.001
Sod2	1.70 [1.10, 2.42]	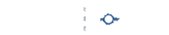	0.001	1.07 [0.73, 1.40]	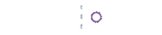	<0.001

Effects are expressed on the log_2_(2^−ΔΔCt^) scale. Confidence intervals were obtained by stratified bootstrap resampling with 10,000 iterations. q values were derived using Benjamini–Hochberg FDR correction. Q, quercetin; HFD, high-fat diet. PIK3R1 denotes the assay label used in the experimental dataset.

## Data Availability

The data supporting the findings of this study are provided within the article, its Supplementary Materials, and the public GitHub repository associated with this work. The repository includes individual raw qPCR Ct values, RT-qPCR normalization and relative-expression datasets, analysis-ready phenotypic data, R scripts used for statistical analysis and figure generation, reproducibility files, and supplementary analytical outputs. The revised Supplementary Materials additionally provide weekly intake summaries, FTO/ACTB technical qPCR quality-control outputs, and shrinkage covariance sensitivity results. The repository is publicly available at https://github.com/alexciencia94/quercetin-hfd-hypothalamus-m6a (accessed on 1 October 2026).

## Supplementary Materials

The following supporting information can be downloaded at: https://www.mdpi.com/article/10.3390/metabo16100773/s1, Table S1: Individual raw Ct values for the 21 target transcripts and the endogenous reference gene ACTB; Table S2: Complete RT-qPCR normalization and relative-expression dataset, including ΔCt, ΔΔCt, 2^−ΔΔCt, and log2-transformed relative-expression values; Table S3: Module PC1 loadings and gene- and module-level robustness analyses; Table S4: Complete treatment-adjusted transcriptional covariance network and robustness classification; Table S5: Weekly group-level food, fluid, and calculated energy-intake values; Table S6: FTO and ACTB technical qPCR quality-control data, including (A) technical-replicate variability, standard-curve efficiency, and melt-peak characteristics, and (B) the FTO and ACTB low-input detection series; Table S7: Ledoit–Wolf shrinkage sensitivity analysis of the treatment-adjusted covariance matrix; Figure S1: Scree plot of the principal component analysis of the 21-gene hypothalamic transcriptional panel; Figure S2: Leave-one-sample-out stability analysis of gene-level FDR-adjusted results; Figure S3: Residual-expression scatterplots for the strongest robust cross-family associations; Figure S4: FTO and ACTB standard-curve and Ct-distribution quality-control plots.

## Abbreviations

ACTB, beta-actin; AGRP, agouti-related peptide; AKT, protein kinase B; AUC, area under the curve; FDR, false discovery rate; FOXO1, forkhead box O1; HFD, high-fat diet; HDL-C, high-density lipoprotein cholesterol; iAUC, incremental area under the curve; IRS1, insulin receptor substrate 1; LDL-C, low-density lipoprotein cholesterol; LEPR, leptin receptor; m6A, N6-methyladenosine; MC4R, melanocortin 4 receptor; OGTT, oral glucose tolerance test; PCA, principal component analysis; PERMANOVA, permutational multivariate analysis of variance; POMC, proopiomelanocortin; Q, quercetin; RT-qPCR, reverse-transcription quantitative polymerase chain reaction; SOCS3, suppressor of cytokine signaling 3.

## Appendix A

**Table A1:** Primer sequences and assay-specific RT-qPCR conditions for the 21 target transcripts and ACTB.

Gene	Forward Primer (5′–3′)	Reverse Primer (5′–3′)	Annealing Temperature (°C)	NCBI RefSeq
*Agrp*	GCAGACCGAGCAGAAGATGT	GCGGTTCTGTGGATCTAGCA	60	NM_033650.1
*Akt2*	ACCCCCAGACCGATATGACA	GGAGAACTGGGGGAAGTGTG	60	NM_017093.1
*Alkbh5*	GGTCTGCTGCCCTACATCTC	CCAACCTGAACAGTGGGGAA	60	NM_001191643.1
*Foxo1*	CAGCCAGGCACCTCATAACA	GTTTGCTGTGCATGTCCAGG	60	NM_001191846.3
*Fto*	GCTCTCCGTGGAACAAAGGA	TCCTCAGATTCTGGCGCATG	60	NM_001039713.1
*Irs1*	GAGTGGCCCCAATGGCTAC	GACCCACCTCCAATGTCAGG	60	NM_012969.2
*Lepr*	CGCCACCGAGTACAACTGAT	GAAGCCCCCTTCAAAGACGA	60	NM_012596.3
*Mc4r*	TTGCTCGCATCCATTTGCAG	TGGTACTGGAGCGCGTAAAA	60	NM_013099.3
*Mettl3*	GCCACTCAAGATGGGGTAGAAA	TAGGAGACCTCGCTTTACCTCA	60	NM_001024794.1
*Mettl14*	TGGAAATAGGAGCGCAGTGG	ACGACAAGGAAAGAGCACCA	60	NM_001399249.1
*Npy*	TGGCCAGATACTACTCCGCT	CTCAGGGCTGGATCTCTTGC	60	NM_012614.2
*Pik3r1*	GAGGATTTGCCCCACCATGA	CTCGCAATAGGTTCTCGGCT	60	NM_013005.3
*Pomc*	CCTCAGAGAGCTGCCTTTCC	CCTGAGCGACTGTAGCAGAA	60	NM_139326.3
*Rbm15*	TGAGTGATGGACAGCGCTTT	TCCGGCGGCTGAATAAAGTT	60	NM_001399561.1
*Rbm15b*	GGAGCATTCGGACCATCGAT	GCATCCAGGCTCTCGTACTG	60	NM_001399320.1
*Socs3*	CGAGAAGATCCCGCTGGTAC	CGACAAAGATGCTGGAGGGT	60	NM_053565.2
*Sod2*	AATCAGGACCCACTGCAAGG	TAGTAAGCGTGCTCCCACAC	60	NM_017051.2
*Virma*	CATGCAAGCAGAACACCCAC	TCGACAGAAGCAAGGTGACC	60	NM_001107915.1
*Wtap*	TCGTCTGGCAAATGGACCAA	GCTCCCCTCCCTGTGAAATC	60	NM_001113542.1
*Ythdc1*	GCTTCAGTTCTCAGTCAGCCT	GTGCCTTAATTGCTGCAGCTT	60	NM_001024756.1
*Zc3h13*	CTCACAGAAGACAGGCAGGG	AGCGCCTTGAACTCTCCTTC	60	NM_001170471.1
*Actb*	GGTGTGATGGTGGGTATGGG	AGGGTCAGGATGCCTCTCTT	60	NM_031144.3

Primer sequences are shown in the 5′→3′ orientation. Gene symbols follow Rattus norvegicus nomenclature. ACTB was used as the endogenous reference gene for RT-qPCR normalization. The PIK3R1 primer pair maps to Rattus norvegicus Pik3r1 (RefSeq NM_013005.3). FTO/ACTB standard-curve, technical-replicate, melt-curve, and low-input quality-control data are provided in Supplementary Table S6 and Figure S4.
